# Exploring the contribution of pro-inflammatory cytokines to impaired wound healing in diabetes

**DOI:** 10.3389/fimmu.2023.1216321

**Published:** 2023-07-27

**Authors:** S. Nirenjen, J. Narayanan, T. Tamilanban, Vetriselvan Subramaniyan, V. Chitra, Neeraj Kumar Fuloria, Ling Shing Wong, Gobinath Ramachawolran, Mahendran Sekar, Gaurav Gupta, Shivkanya Fuloria, Suresh V. Chinni, Siddharthan Selvaraj

**Affiliations:** ^1^ Department of Pharmacology, SRM College of Pharmacy, SRM Institute of Science and Technology, Kattankulathur, Tamil Nadu, India; ^2^ Jeffrey Cheah School of Medicine and Health Sciences, Monash University, Jalan Lagoon Selatan, Bandar Sunway, Petaling Jaya, Selangor, Malaysia; ^3^ Center for Transdisciplinary Research, Department of Pharmacology, Saveetha Dental College, Saveetha Institute of Medical and Technical Sciences, Saveetha University, Chennai, Tamil Nadu, India; ^4^ Faculty of Pharmacy, AIMST University, Bedong, Kedah, Malaysia; ^5^ Faculty of Health and Life Sciences, INTI International University, Nilai, Malaysia; ^6^ Department of Foundation, RCSI & UCD Malaysia Campus, Jalan Sepoy Lines, Georgetown, Pulau Pinang, Malaysia; ^7^ School of Pharmacy, Monash University Malaysia, Subang Jaya, Selangor, Malaysia; ^8^ School of Pharmacy, Suresh Gyan Vihar University, Jagatpura, Mahal Road, Jaipur, India; ^9^ Uttaranchal Institute of Pharmaceutical Sciences, Uttaranchal University, Dehradun, India; ^10^ Department of Biochemistry, Faculty of Medicine, Bioscience, and Nursing, MAHSA University, Jenjarom, Selangor, Malaysia; ^11^ Department of Periodontics, Saveetha Dental College and Hospitals, Saveetha Institute of Medical and Technical Sciences, Saveetha University, Chennai, India; ^12^ Faculty of Dentistry, AIMST University, Semeling, Kedah, Malaysia

**Keywords:** wound healing, pro-inflammatory, diabetes mellitus, cytokines, therapeutic approach

## Abstract

**Background:**

Impaired wound healing is the most common and significant complication of Diabetes. While most other complications of Diabetes have better treatment options, diabetic wounds remain a burden as they can cause pain and suffering in patients. Wound closure and repair are orchestrated by a sequence of events aided by the release of pro-inflammatory cytokines, which are dysregulated in cases of Diabetes, making the wound environment unfavorable for healing and delaying the wound healing processes. This concise review provides an overview of the dysregulation of pro-inflammatory cytokines and offers insights into better therapeutic outcomes.

**Purpose of review:**

Although many therapeutic approaches have been lined up nowadays to treat Diabetes, there are no proper treatment modalities proposed yet in treating diabetic wounds due to the lack of understanding about the role of inflammatory mediators, especially Pro-inflammatory mediators- Cytokines, in the process of Wound healing which we mainly focus on this review.

**Recent findings:**

Although complications of Diabetes mellitus are most reported after years of diagnosis, the most severe critical complication is impaired Wound Healing among Diabetes patients. Even though Trauma, Peripheral Artery Disease, and Peripheral Neuropathy are the leading triggering factors for the development of ulcerations, the most significant issue contributing to the development of complicated cutaneous wounds is wound healing impairment. It may even end up with amputation. Newer therapeutic approaches such as incorporating the additives in the present dressing materials, which include antimicrobial molecules and immunomodulatory cytokines is of better therapeutic value.

**Summary:**

The adoption of these technologies and the establishment of novel therapeutic interventions is difficult since there is a gap in terms of a complete understanding of the pathophysiological mechanisms at the cellular and molecular level and the lack of data in terms of the assessment of safety and bioavailability differences in the individuals’ patients. The target-specific pro-inflammatory cytokines-based therapies, either by upregulation or downregulation of them, will be helpful in the wound healing process and thereby enhances the Quality of life in patients, which is the goal of drug therapy.

## Graphical abstract

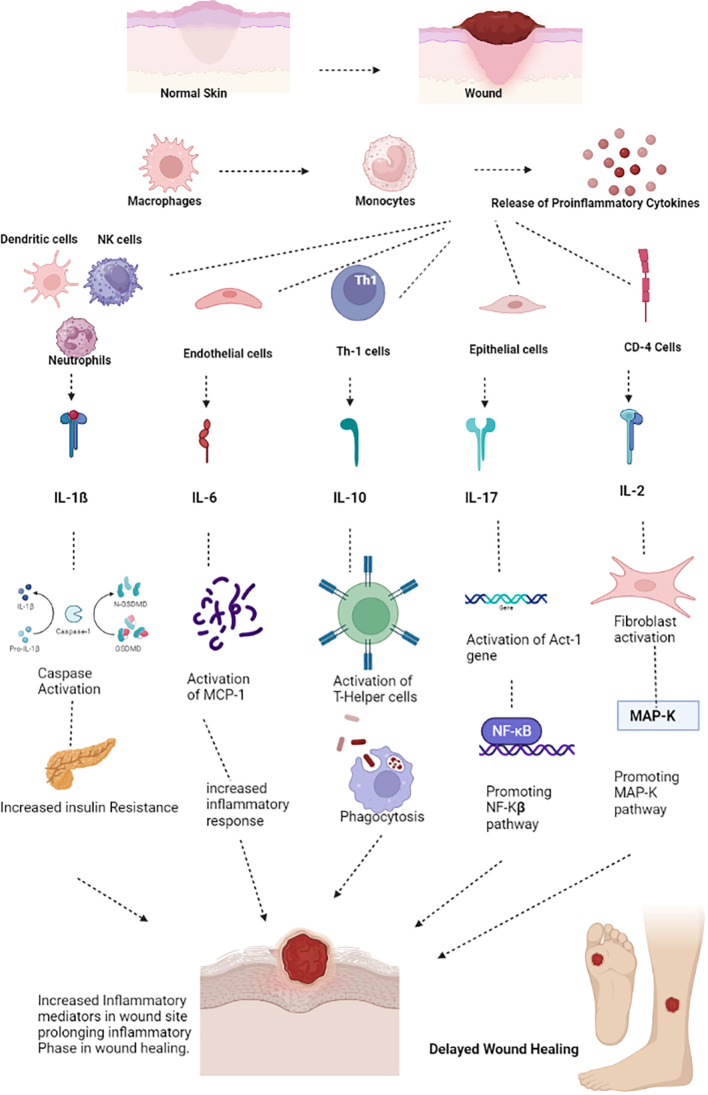

## Highlights

- Impaired wound healing is a significant complication of Diabetes that remains a burden on patients as it can cause pain and suffering.
- Pro-inflammatory cytokines play a critical role in wound closure and repair, but their dysregulation in Diabetes creates an unfavorable environment for healing and delays the wound healing processes.
- This concise review offers insights into better therapeutic outcomes by providing an overview of the dysregulation of pro-inflammatory cytokines in diabetes mellitus.

## Background

Diabetes is the most common metabolic disorder and is more prevalent than other metabolic disorders. It is a chronic condition characterized by marked elevations in blood glucose levels, known as hyperglycemia. Diabetes is a growing concern, with 2.6 million individuals diagnosed yearly; approximately 4% of the population struggles with this disorder. Type-2 Diabetes Mellitus affects an estimated 2.3 million people, while type-I DM affects around 0.3 million (1). Diabetes is one of the leading causes of death worldwide. A current statistical survey conducted by the National School of Economics stated that Diabetes is one of the ten leading causes of death, which estimates about 6.7 million deaths (2). The global economic burden of Diabetes Mellitus, along with its complications, is expected to reach 2.1 trillion in upcoming years, which equates to about 10% of the total National Health Budget. Diabetes and its complications have an impact on the financial status of the patient (3).

Although complications of Diabetes Mellitus are mostly reported after years of diagnosis, impaired wound healing among Diabetes patients is the most severe and critical complication. Even though trauma, peripheral artery disease, and peripheral neuropathy are the leading triggering factors for the development of ulcerations, the most significant issue contributing to the development of complicated cutaneous wounds is impaired wound healing, which may even result in amputation (4). Dysregulation or defects in the microcirculation, which are associated with peripheral artery disease and peripheral neuropathy, contribute to or result in impaired wound healing in Type-II Diabetes Mellitus. In individuals with type 1 diabetes, the high blood sugar levels can negatively affect each of these stages, leading to impaired wound healing. One of the main complications of type 1 diabetes is poor blood circulation. High blood sugar levels can damage blood vessels, reducing blood flow to the affected area. This diminished blood supply means that essential nutrients and oxygen required for proper wound healing may not reach the wound efficiently, slowing down the healing process. Moreover, individuals with type 1 diabetes often have weakened immune systems, making them more susceptible to infections. This is due to high blood sugar levels impairing the function of immune cells responsible for fighting off bacteria and other pathogens. As a result, wounds in people with type 1 diabetes are more prone to infections, which can further delay the healing process. Additionally, the long-term effects of high blood sugar levels can cause nerve damage, known as diabetic nephropathy. This condition can lead to a loss of sensation in the affected area, making it difficult for individuals to detect injuries or wounds. Delayed wound detection and lack of proper care can result in further complications and hinder the healing process (5). Both type 1 and type 2 diabetes can lead to poor blood circulation due to high blood sugar levels and blood vessel damage. Reduced blood flow to the wound site can hinder the delivery of oxygen and nutrients necessary for proper wound healing. Although there are several therapeutic approaches in treating Diabetes and its other complications, there are no proper treatment modalities proposed yet to treat non-healing diabetic wounds due to the lack of understanding about the underlying pathophysiological mechanisms of the role of inflammatory mediators, especially pro-inflammatory mediators such as cytokines, in the wound healing process. This review mainly focuses on this issue.

## Mechanisms involved in the healing of wounds

### The wound healing

When tissue integrity is compromised, an effective and intricate process called wound healing begins (6). The four stages of healing—hemostasis, inflammation, proliferation, and remodeling- are the most intricate processes and include several steps (7).

#### Hemostasis

Platelets are essential for hemostasis, the initial phase of tissue healing. As circulating platelets come into contact with the collagen of the wounded tissue, they become activated, gather, and stick to the damaged endothelium (8). When coagulation begins, fibrinogen is transformed into fibrin, forming the thrombus and the temporary extracellular matrix. When platelets are activated, they release proteins that cause neutrophils and monocytes to migrate and adhere to one another, as well as several growth factors, such as transforming growth factor-ß (TGF-ß) and platelet-derived growth factor (PDGF), that facilitate wound healing (9).

#### Inflammatory phase

**Figure 1:**
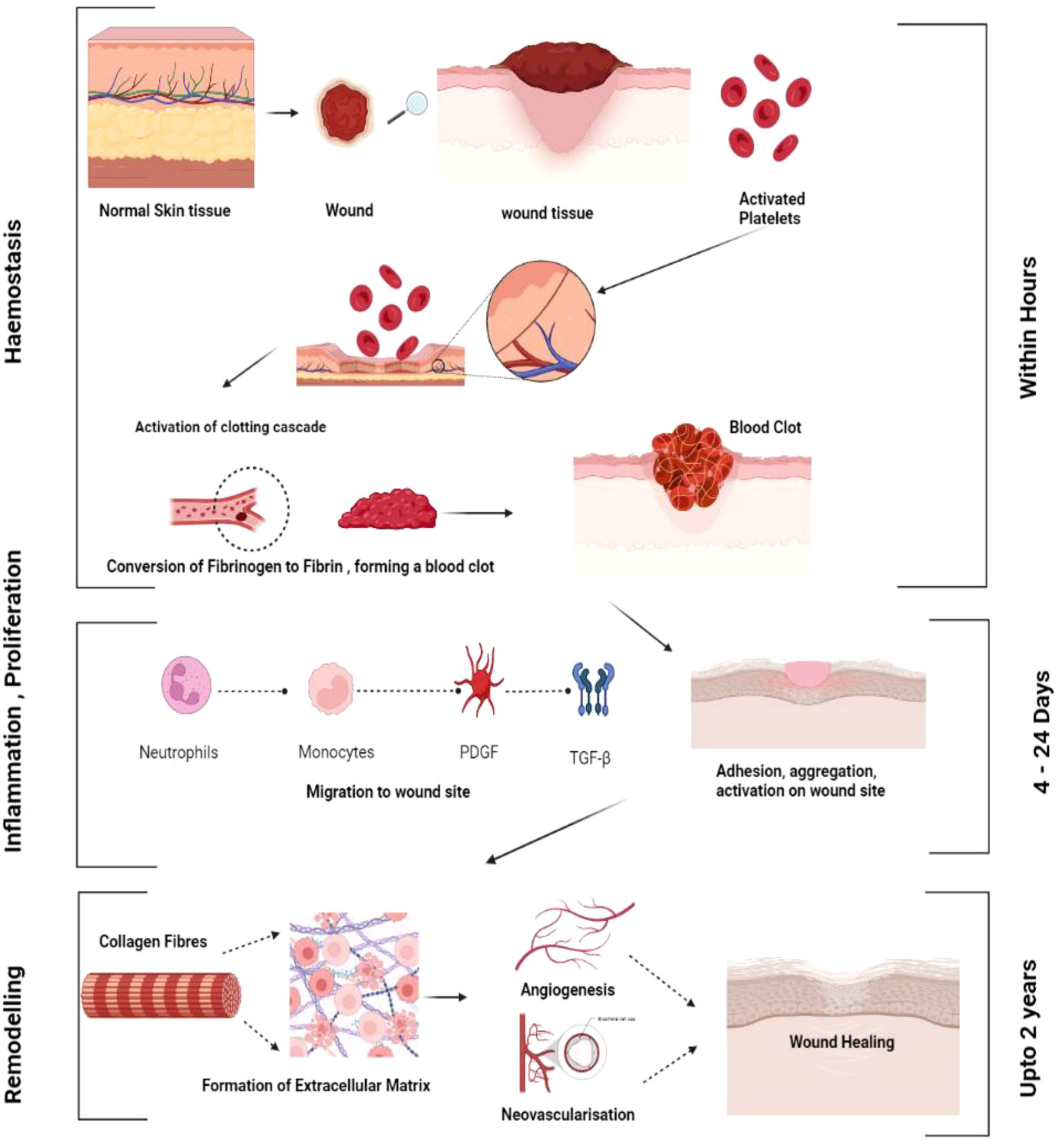
General mechanism of wound healing. Various phases of wound healing haemostasis, inflammation, proliferation and remodelling.

As soon as an injury occurs, the inflammatory phase of wound healing begins as inflammatory cells infiltrate the wound site. The first cells to devour the injured tissue are neutrophils. When sticky molecules on the vascular endothelial surface near the wounded tissue are activated, neutrophils adhere to the endothelium. Then, neutrophils advance further into the tissue space through damaged capillaries or gaps between endothelial cells, a process known as diapedesis (10). Neutrophils are essential for tissue debridement and infection control. They also generate a variety of growth factors that encourage cell development and proteases that degrade the extracellular matrix, making them involved in the wound-healing process (11). When circulating monocytes enter the tissue area, they become mature macrophages (M2 Macrophages), creating an inflammatory response. Through phagocytosis, pro-inflammatory or activated macrophages (M1 macrophages) clear the wound of germs, foreign objects, apoptotic neutrophils, and injured tissue fragments (12). They also produce a range of cytokines and mediators that promote inflammation. Local mast cells also rapidly react to tissue damage and are crucial for wound healing (**Figure 1**). Mast cell degranulation produces proteinases that break down the extracellular matrix and several cytokines that stimulate neutrophil recruitment. T-lymphocytes arrive at the wound site in the late stages of inflammation and appear to coordinate tissue remodeling (13).

#### Proliferative phase

The wound enters the proliferative phase as inflammation subsides and macrophages change to an alternate activated or anti-inflammatory phenotype. The transition of macrophages from a pro-inflammatory phenotype to an alternative activated or anti-inflammatory phenotype plays a crucial role in the shift from the inflammatory phase to the proliferative phase of wound healing. Complex molecular and cellular mechanisms govern this transition. During the inflammatory phase, macrophages are primarily of the pro-inflammatory M1 phenotype. These M1 macrophages produce and release pro-inflammatory cytokines such as interleukin-1 beta (IL-1β), tumor necrosis factor-alpha (TNF-α), interleukin-6 (IL-6), and interleukin-8 (IL-8). They are involved in phagocytosis, secretion of chemokines, and initiating the inflammatory response. However, as the inflammatory response subsides, the wound microenvironment undergoes changes that trigger the transition of macrophages to an anti-inflammatory or alternative-activated M2 phenotype. Several signaling molecules and cytokines contribute to the shift from M1 to M2 phenotype. Transforming growth factor-beta (TGF-β) and IL-10 are key cytokines promoting the M2 phenotype. These cytokines can be produced by other immune cells, such as regulatory T cells and Th2 lymphocytes, as well as by the macrophages themselves in an autocrine manner. Transcription factors play a critical role in regulating gene expression and macrophage polarization. Hypoxia-inducible factor-1 alpha (HIF-1α) and peroxisome proliferator-activated receptor-gamma (PPAR-γ) are two key transcription factors involved in promoting the shift to the M2 phenotype. Hypoxia, which is prevalent during the early stages of wound healing, can stabilize and activate HIF-1α, promoting the expression of M2-associated genes.

Macrophages have a crucial role in all stages of wound healing, especially proliferation and remodeling. Pro-inflammatory macrophages, also known as “M1” macrophages, primarily invade the site of damage to rid it of pathogens, foreign bodies, and dead cells. During the healing process of acute wounds, the local macrophage population changes from being mostly pro-inflammatory (M1) to anti-inflammatory (M2). Pro-inflammatory macrophages (M1) first invade the site of injury and eliminate pathogens, foreign debris, and dead cells. Cytokines that are anti-inflammatory M2 encourage cell migration and proliferation of keratinocytes, fibroblast, and endothelial cells to repair the dermis, epidermis, and vascular system. M2 macrophages have serious subsets such as M2a, M2b, and M2c that are pivotal in the proliferative phases of wound healing. IL-4/IL-13 stimulate M2a macrophages. Collagen precursors are produced by M2a macrophages and thereby stimulate the production of fibroblasts in wound healing. The proliferative phase of wound healing necessitates the production of the extracellular matrix (ECM), which is facilitated by M2a macrophages, and PDGF, which is crucial for the process of angiogenesis and is highly secreted by M2a macrophages. M2b macrophages release IL-6, IL-10, TNF, and express high levels of iNOS and produce MMPs, but they also dampen inflammation by upregulating IL-10 synthesis. M2c is stimulated by IL-10 and TGF-ß and produces high amounts of IL-1ß, MMP-9, IL-10, and TGF-β and significantly low amounts of IL-12. M2c engaged in the remodeling of the vascular structure and extracellular matrix (14).

Growth factors, such as vascular endothelial growth factor (VEGF) and TGF-β, which promote cell proliferation and protein synthesis, are also secreted by anti-inflammatory macrophages (M2 macrophages), along with a range of protease inhibitors, proteases, and anti-inflammatory mediators (15). Granulation tissue begins to replace the interim matrix. Using the intermediate matrix as a scaffold, growth factors released by macrophages promote fibroblast migration into the wound (16). The fibroblasts proliferate and begin producing extracellular matrix elements like collagen (fibroplasia). The development of new capillaries through angiogenesis enhances fibroblast growth. New blood vessels must form from an existing capillary network for the rapidly reproducing cells within the healing wound to receive oxygen and nutrients (17). On the other hand, vascularization is the process of generating new blood vessels from scratch by luring endothelial progenitor cells (also known as EPCs) from the bone marrow. A population of adult stem cells known as EPCs can differentiate into epithelial cells in response to tissue ischemia, promoting endothelial regeneration and neovascularization (18).

Angiogenesis is a dynamic, managed, and regulated process, and its major drivers include proangiogenic chemicals such as fibroblast growth factor-2 (FGF-2) and VEGF, as well as anti-angiogenic compounds that affect endothelial cells. FGF-2 appears to be released due to tissue disintegration during the first three days of wound healing, while VEGF release is mostly prompted by tissue hypoxia over the next three days (19). During the first proliferative phase, a microvascular network of fresh capillaries develops across the granulation tissue. Towards the conclusion of the healing process, blood vessel density declines (20). The proliferation of new capillaries gives the new connective tissue a characteristic granular look, earning it the name granulation tissue. When granulation tissue forms, keratinocytes migrate from the edges of the wound or around skin appendages to the new matrix and re-epithelialize. The incision is covered or epithelialized, and the granulation tissue is covered (21). Some growth factors released from the injured epidermis and encourage the proliferation of epithelial cells include endothelial growth factor (EGF), keratinocyte growth factor, and FGF-2 (22).

#### Remodeling phase

The remodeling phase of wound healing starts two to three weeks after the initial injury, and granulation tissue eventually develops into scar tissue. The density of blood vessels decreases, and collagen is organized and modified. During the remodeling phase, continuous collagen formation and breakdown occur and are principally kept in balance by the activity of matrix metalloproteinases (23). The tensile strength of the wound increases due to cross-linking, which is facilitated by the closer spacing of newly synthesized collagen fibers across tension lines. Wound contraction is an additional alternative in which myofibroblasts reduce the size of the wound by drawing the margins of the wound together (24).

## Pathophysiology of diabetic wound healing

Open wounds that do not heal are referred to as non-healing wounds. Chronic ulcers are those that do not heal after a period of 12 weeks. Chronic diabetic wounds start off like acute wounds, but the healing process is slowed down and interrupted at several points along the way. As a result, the usual acute ulcer repair is unsuccessful. The healing of wounds depends on several growth factors, including cytokines, proteases, cells, and extracellular elements (25). In diabetic patients, hyperglycemia, chronic inflammation, micro- and macro-circulatory dysfunction, hypoxia, autonomic and sensory neuropathy, and poor neuropeptide signaling all affect the healing of wounds (26). Advanced glycation end products and non-enzymatic glycation of proteins, such as collagen, can result in hyperglycemia (27). The solubility of the extracellular matrix is decreased by these end products, which also maintain the inflammatory changes seen in diabetes (28).

Non-healing wounds occur when there is a deviation from the normal physiological wound healing mechanism, making it a pathological condition. Diabetes and diabetic wounds bypass specific mechanisms in the wound-healing process and impact all three phases of wound-healing mechanisms (29). Firstly, in the inflammation phase, there is an increased infiltration of inflammatory cells in and around the dermis and vessel walls, elevating inflammatory cytokines such as IL-6, IL-1, and TNF-α, which eventually decreases the expression of various growth factors such as TGF-β following a negative feedback mechanism (30). On the other hand, in the proliferative phase, impaired angiogenesis and vasculogenesis eventually immobilize the migration of inflammatory cells that are involved in tissue repair (31). In the remodeling phase, there is an increase in the levels of MMP, which, in turn, degrades the extracellular matrix, which plays an inevitable role in the wound-healing process (20). The molecular mechanisms of wound healing mainly involve three important pathophysiological mechanisms: inflammation, tissue hypoxia, and the extracellular matrix.

### Inflammation

Experimental and clinical evidence suggests that, unlike normal wound healing, diabetic wounds do not follow the usual sequence of wound healing mechanisms. There is a deviation from normal mechanisms, with overexpression of IL-1ß, TNF-α, and Monocyte Chemoattractant Proteins (MCP-1) in serum (30). Macrophages are the primary sources of pro-inflammatory cytokines, and macrophage dysfunction is a major pathophysiological mechanism in impaired wound healing in Diabetes. Macrophages play a critical role in the inflammatory phase, where the initiation of wound healing begins by helping in the formation of new tissues. Recent studies suggest the presence of myeloid cells in the wound site, which recruit more pro-inflammatory cytokines, resulting in delayed wound healing (32). Besides macrophages, increased expression of neutrophils also appears to have a harmful effect on wound healing mechanisms, as neutrophil infiltration is a primary process in prolonged inflammation. Other studies support the idea that neutrophil PAD-4 is highly expressed in patients with hyperglycemia and favors delayed wound healing. Treatment targets should focus on PAD-4 deficiency, which may improve wound healing in diabetic patients.

### Tissue hypoxia

Hypoxia, or a deficiency in oxygen supply, can significantly affect various cellular processes, including the plasticity of circulating monocytes involved in wound healing. Monocytes are white blood cells that play a crucial role in immune responses and tissue repair. During wound healing, monocytes are recruited to the injured site and differentiate into macrophages, highly versatile cells capable of performing various functions, such as phagocytosis (engulfing and eliminating foreign substances), producing growth factors, and regulating inflammation. This process is known as monocyte/macrophage plasticity.

Hypoxia can affect the expression of various matrix metalloproteinases (MMPs) by monocytes/macrophages. MMPs are enzymes in remodeling the extracellular matrix (ECM), providing structural support to tissues. Hypoxia-induced MMPs can facilitate the breakdown and remodeling of the ECM during wound healing. Hypoxia stimulates the release of angiogenic factors, such as VEGF and platelet-derived growth factor (PDGF), by monocytes/macrophages. These factors promote the formation of new blood vessels (angiogenesis), essential for delivering oxygen and nutrients to the wound site. Hypoxia can modulate the cellular plasticity of circulating monocytes, promoting their recruitment, altering their phenotype, and enhancing their functional properties. These changes contribute to the wound-healing process by regulating inflammation, angiogenesis, ECM remodeling, and tissue repair (9).

The “angiogenic switch” refers to transitioning from limited angiogenesis (formation of new blood vessels) to a highly angiogenic state during certain physiological or pathological conditions. In the proliferative phase of wound healing, the angiogenic switch is crucial in supplying oxygen and nutrients to the healing tissue. During the early stages of wound healing, such as the inflammatory phase, there is initially limited angiogenesis due to vasoconstriction and the formation of blood clots to prevent excessive bleeding. However, as the wound progresses into the proliferative phase, which involves granulation tissue formation and re-epithelialization, the need for increased blood vessel formation arises. Hypoxia, or low oxygen levels, is a key trigger for the angiogenic switch during the proliferative phase of wound healing. When tissues are exposed to hypoxia, various molecular and cellular responses are activated to stimulate angiogenesis. The angiogenic switch, regulated by hypoxia-induced signaling pathways, plays a pivotal role in initiating and promoting angiogenesis during the proliferative phase of wound healing. The balance between proangiogenic and anti-angiogenic factors is dynamically regulated to ensure appropriate blood vessel formation and tissue revascularization.

Clinically, hypoxia refers to increased oxygen consumption by our body beyond its needs or decreased oxygen supply. Hypoxia tends to maintain the levels of Hypoxia Inducible Factor (HIF-1), which regulates numerous cellular processes, including angiogenesis and proliferation, and as a result, can cause delayed wound healing (33). However, the expression of HIF-I genes can lead to impaired angiogenesis and proliferation, resulting in delayed wound healing. Conversely, the expression of HIF-α genes can improve the wound-healing process.

### Extracellular matrix

**Figure 2:**
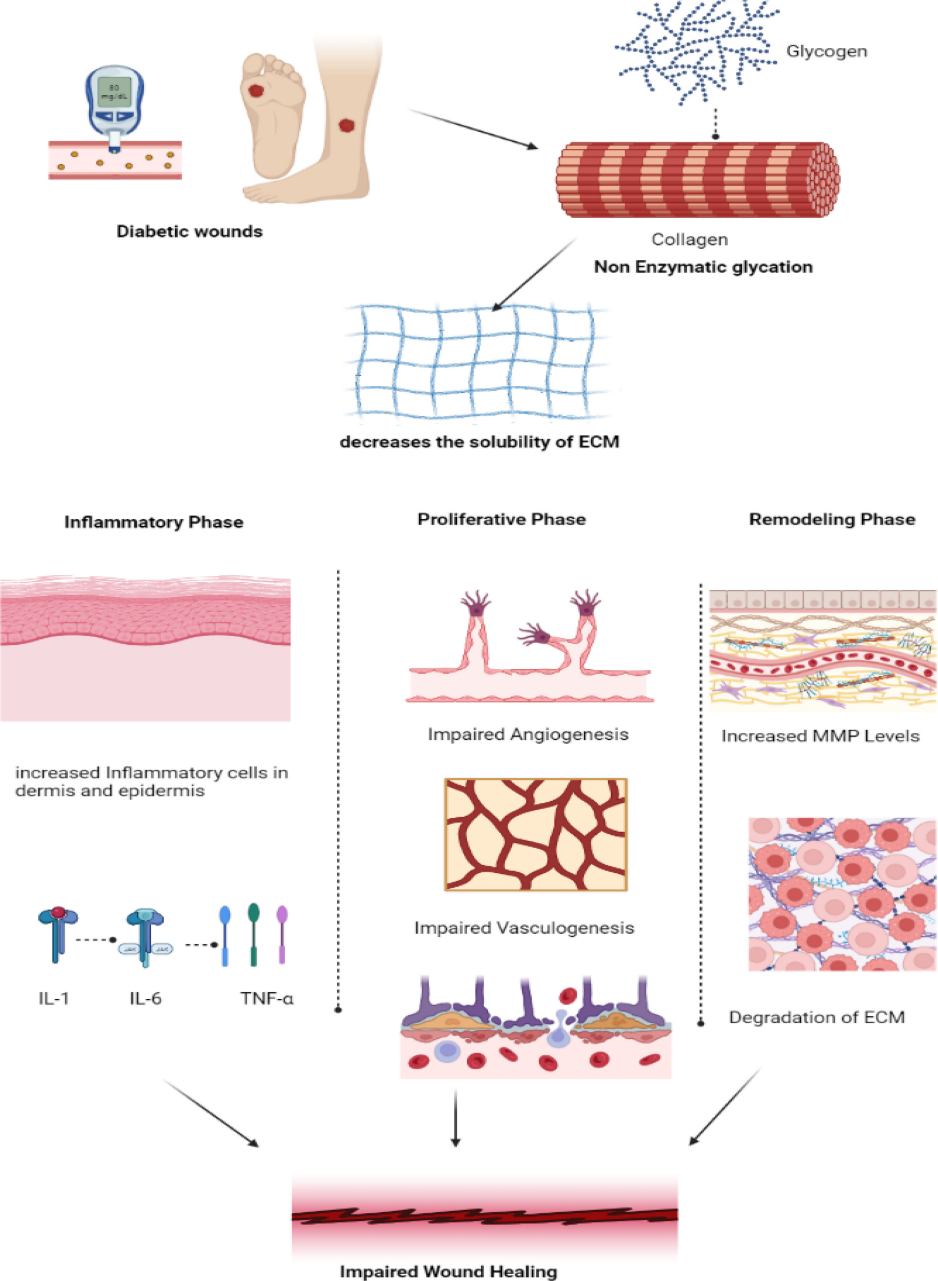
Pathogenesis of impaired wound healing in diabetes. In diabetic wounds, there is an impairment in the collagen synthesis thereby decreasing the solubility of the Extracellular matrix promoting increased inflammatory response by the release of pro-inflammatory cytokines IL-1, IL-6 and TNF-α in inflammatory phase. Whereas in proliferative phase, impairment in angiogenesis and vasculogenesis and in remodeling phase increased MMP leads to degradation of ECM thereby resulting in impaired wound healing. (IL – Interleukin, TNF- Tumor Necrosis Factor, MMP- Matrix Metalloproteinase, ECM- Extracellular Matrix).

The Extracellular matrix is a key environmental component in healing diabetic wounds, as it acts as a dynamic scaffold for cells and facilitates tissue development and regeneration (**Figure 2**). Fibroblasts aid in the rapid formation and deposition of the Extracellular matrix, which promotes not only cellular-level binding but also improves the process of regeneration and angiogenesis (34). The cohesion of the Extracellular matrix depends mainly on endopeptidases such as Matrix Metallo Proteases (MMP) and Tissue Inhibitors of Metalloproteinases (TIMP). In the case of inflammation in diabetic wounds, there is an over-expression of MMP and an under-expression of TIMP. MMP-9 plays a major role in cell migration and proliferation, aiding the wound-healing process by promoting the sequence of events of cell migration, proliferation, and collagen synthesis, thereby promoting wound healing (35).

## Cytokines in wound healing

**Figure 3:**
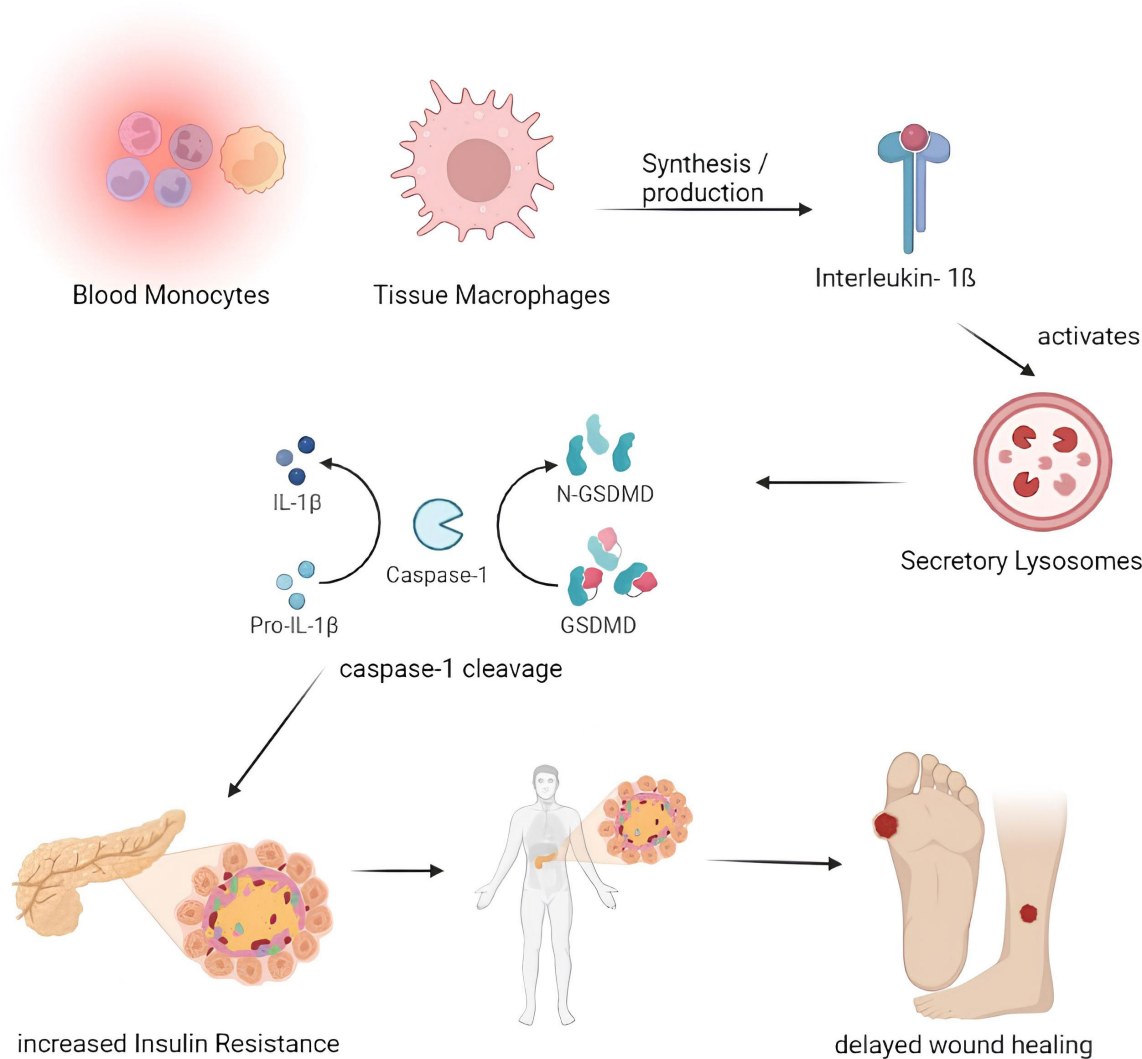
Pathogenesis of impaired wound healing involving IL-1β. Blood Monocytes and Tissue Macrophages synthesize the IL-1β which activates the secretory lysosomes and resulting in the Caspase- 1 cleavage through IL-1β and GSDMD which in turn results with increased insulin resistance thereby delays the process of wound healing. (IL – Interleukin, GSDMD - protein gasdermin D).

The most significant inflammatory chemicals, cytokines, are produced by tissue macrophages and blood monocytes. When there is an infection, leukocytes produce cytokines, and interleukins are produced toward the target cell. These cytokines cause an increase in IL levels by activating the signals within the target cells. This increase in IL levels can lead to Diabetes and result in a refractory repair and the onset of insulin resistance in patients with DM (**Figure 3**).

### IL- 1β

Both blood monocytes and tissue macrophages produce IL-1β, a significant inflammatory cytokine. Following activation of the NALP3 inflammasome by caspase-1, IL-1ß cytokine is activated in the secretary lysosome by caspase-1 cleavage. In addition to inflammasome activation, IL-1β promotes inflammation by boosting bone marrow-derived leukocyte mobilization and liver-derived acute-phase protein production (36). Increased IL-1β has been associated with developing insulin resistance and slowed wound healing in Diabetes. IL-1β levels are elevated in human diabetic foot ulcers and decrease when the ulcers heal (37). Diabetic human and db/db mouse wound macrophages exhibit elevated IL-1β and NALP3 inflammasome components during the first ten days of healing, and inhibiting the inflammasome was associated with better wound healing in these models (38). Anakinra (Kineret), an IL-1β receptor antagonist, is frequently prescribed to treat rheumatoid arthritis (RA) (39). In normal mice, Anakinra has been shown to be effective at lowering the levels of TNF-α and IL-6 proteins in wound tissue during the first 48 hours following wound healing. Thus, inhibiting IL-1β function in diabetic wounds using neutralizing antibodies or a receptor antagonist may promote wound healing (40).

### IL-6

**Figure 4:**
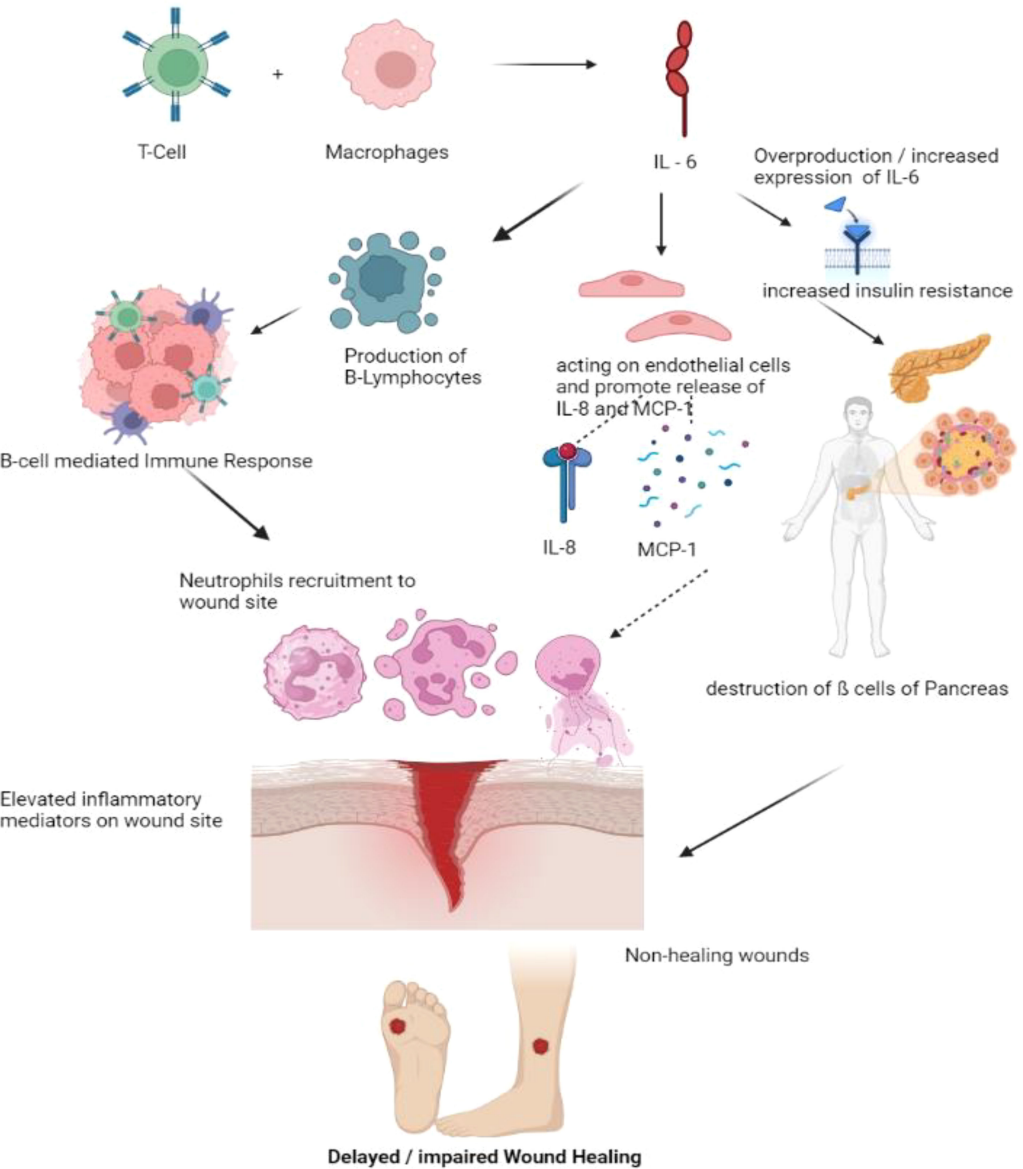
Pathogenesis of impaired wound healing involving IL-6. T- Cells and Macrophages secretes the IL-6. Over production or elevated IL-6 levels increases the insulin resistance, Synthesized IL-6 may also generate the B-cell mediated immune response, recruiting more neutrophils to the wound site and also promote the release of IL-8 and MCP thereby recruiting more inflammatory cells to wound site delaying the process of wound healing. (IL – Interleukin, MCP – Monocyte Chemoattractant Protein).

T cells and macrophages secrete the cytokine IL-6, essential for host defense. In addition to promoting the growth of B lymphocytes, IL-6 stimulates the production of neutrophils in the bone marrow and the release of acute-phase proteins from the liver (41). Additionally, it affects leukocyte recruitment by causing endothelial cells to secrete IL-8 and MCP-1. Increased levels of IL-6 are related to insulin resistance and inflammation in β-cells in Diabetes. Rabbits fed the toxic glucose analog alloxan monohydrate have significantly higher levels of IL-6 wound expression than controls (42). They also had significantly delayed wound healing. In macrophages derived from normal mice, hyperglycemia tends to greatly increase IL-6 expression dose-dependently; this finding is likewise supported by mice treated with streptozotocin (STZ) and db/db mice’s isolated macrophages (43). Compared to diabetic patients without foot ulcers, acute-phase proteins in the blood and IL-6 were considerably higher in diabetic individuals with foot ulcers. The increased IL-6 expression seen in diabetic wounds appears to correlate highly with glucose levels and wound chronicity. Models of corneal alkali burns have demonstrated that inhibiting the IL-6 receptor reduces inflammation, suggesting a possible usefulness in its application in chronic wounds (44). The anti-IL-6 receptor antibody tocilizumab (Actemra) is proven to effectively lower blood glucose levels in people with Type 2 diabetes (29). If IL-6 antibody therapy fails to reduce its expression in diabetic wound healing, natural therapies may be an alternative in mice given STZ. Injections over a period of seven weeks, curcumin, one of the primary components of turmeric, has been demonstrated to drastically lower circulating plasma levels of IL-6 (45). In contrast, employing curcumin-loaded nanofibers improved wound healing in an STZ-induced diabetes model and decreased the quantity of IL-6 produced *in vitro* from lipopolysaccharide-activated macrophages (46). These findings imply that decreasing levels of circulating IL-6, whether through antibody-mediated or natural means, may be an essential tool in treating diabetic wounds (**Figure 4**).

### IL -10

IL-10 is an inflammatory cytokine that is synthesized by macrophages, dendritic cells, T-helper cells, and activated T-helper cells (47). IL-10R is expressed most in immune cells, especially macrophages, where it inhibits antigen presentation and promotes phagocytosis. IL-10R signaling is necessary for the expansion of anti-inflammatory macrophages that regulate mucosal defense in both mice and humans (48).

Type 2 diabetes, obesity, and decreased blood IL-10 levels are all linked (49). Wound-derived macrophages isolated from db/db mice express less IL-10 protein than those from db/+ mice over a period of seven days. Moreover, STZ-injected animals exhibited a significant decrease in tissue IL-10 protein levels than did control rats seven days after injury (50). IL-10 expression is reduced in human diabetic foot ulcers, particularly in endothelial cells and keratinocytes along wound borders (51). Recently, the topical application of curcumin to wounds of STZ-treated rats resulted in elevated IL-10 mRNA and protein levels, improved granulation tissue formation, and enhanced wound contraction (52, 53). As there is an increase in IL-10 in diabetic wounds, inhibiting IL-10 may present an intriguing possibility for enhancing healing.

### IL – 17

IL-17 has a potential role in the pathophysiological manifestations of wound healing and could be used as a potential novel target for wound healing therapy (54). The IL-17 cytokine family consists of 5 subtypes: IL-17A, IL-17C, IL-17F, IL-17B, and IL-17E (55). Among these, IL-17B and IL-17E have not been well recognized, as their pathways have not been studied yet. These subfamilies of cytokines readily act upon the keratinocytes and promote the expression of chemokines, which eventually start recruiting more neutrophils to the wound site for the process of neutrophil infiltration, diapedesis, and thereby initiating wound healing (56).

**Figure 5:**
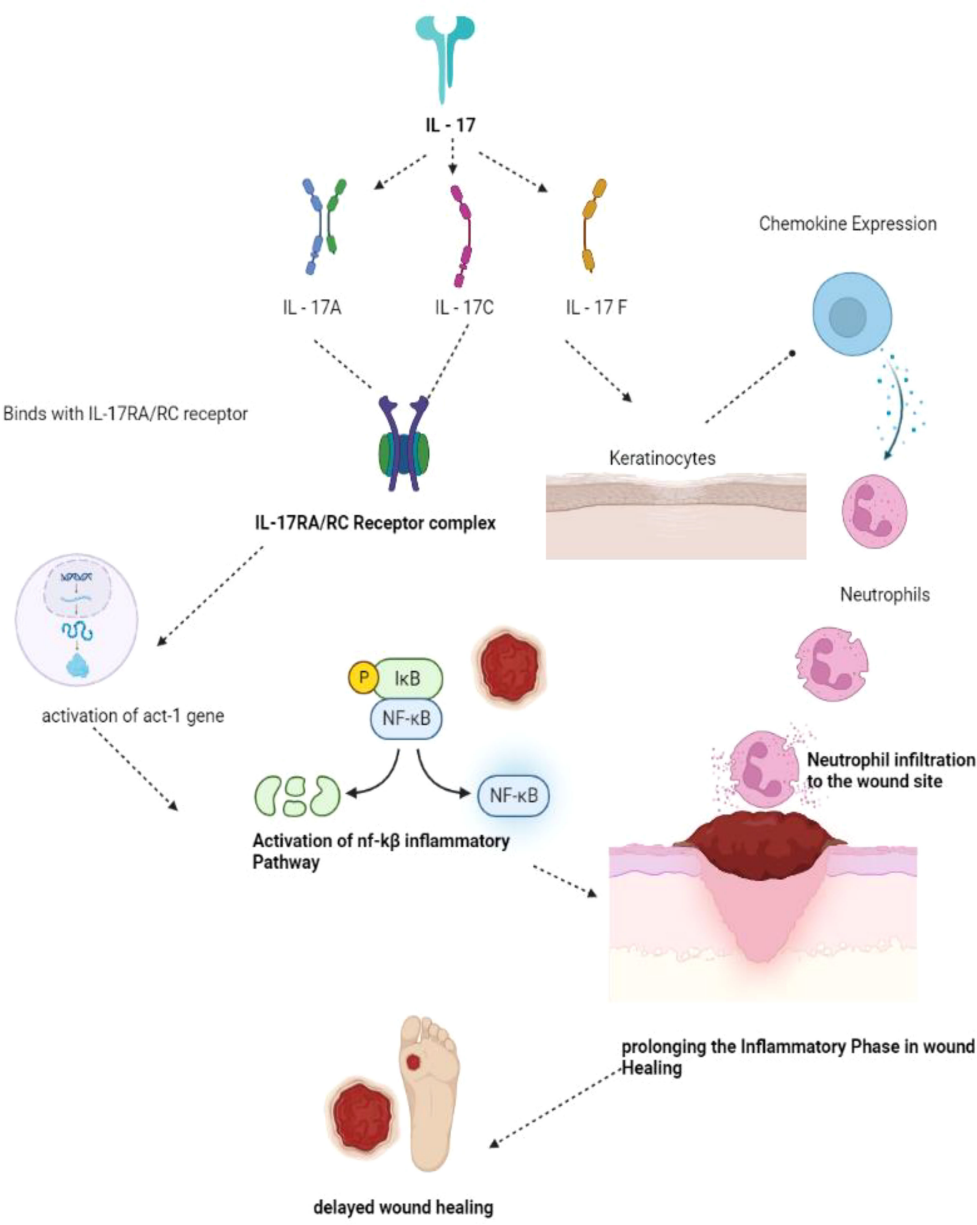
IL-17 mechanism of wound healing. IL- 17 has been sub classified as IL-17A, 17C and 17F. IL-17A and 17C binds with IL-17RA/RC forming the receptor complex in turn activates the act-1 gene activates NF-kB pathway of inflammation thereby prolonging the inflammatory phase delaying the wound healing phase. IL-17F acting on the keratinocytes produces neutrophils prolonging inflammatory phase and delaying the process of wound healing. (IL – Interleukins, NF-kB - Nuclear factor kappa B).

The pathophysiological mechanism involved in IL-17 involves the following sequence of events. Firstly, cells of both innate and adaptive immunity, as well as a wide range of non-hematopoietic cells, activate the release of Transforming Growth Factor-β and several pro-inflammatory cytokines such as IL-1β, IL-6, and IL-23 (57). IL-1β promotes the release of IL-17C from the epithelial cells, while IL-23 stimulates the production of IL-17A and IL-17F from the T-Helper cells, which are the natural killer cells (58). Upon release of the IL-17 family (IL-17A, IL-17C, IL-17F), IL-17A and IL-17F bind with the IL-17RA/RC receptor, and IL-17 binds with the IL-17RA/RE receptor, forming a receptor complex. Upon binding, IL-17A, IL-17C, and IL-17F prompt conformational changes and activate the Act-1 gene, which, in turn, activates Mitogen-Activated Protein Kinase (MAPK) and Nuclear Factor-kβ signaling cascades (59). These signaling pathways lead to the expression of several peptides, chemokines, and cytokines, forming an inflammatory environment preferable for wound healing. Thus, in brief, upregulation of the IL-17-based signaling pathway may result in prolonged inflammation and delayed wound healing (**Figure 5**). IL-17 has some favorable roles in wound healing through the proliferation of keratinocytes and the release of antimicrobial peptides that could be used as a potential target in wound healing (60). IL-17-based therapeutics are now used in the healing of psoriatic wounds where there seems to be upregulation of IL-17A. The IL-17A receptor antagonist drug Secukinumab, an active monoclonal antibody drug, shows significant therapeutic outcomes in healing psoriatic wounds (61).

### IL-2

IL-2 plays a significant role in wound healing as it is involved in the T-cell-mediated immune response and promotes wound healing by improving the strength of healed skin, thereby promoting wound closure (62). Interestingly, most cell types, including skin cells and fibroblasts, express the IL-2 receptors, making them a suitable potential target for site-specific therapies in wound healing (63). IL-2 signaling is a complex cascade of events where IL-2 is produced by various cell types, such as Tetrahydro curcumin (THC), Cytotoxic T Lymphocytes (CTLs), Macrophages, Fibroblasts, and Keratinocytes. IL-2 receptors consist of three sub-units: IL-2Rα, IL-2Rβ, and IL-2Rγ. These subunits may have different affinities toward the specific receptors for IL-2 binding (64). IL-2 cell signaling involves the JAK-STAT pathway, where IL-2 readily binds to the receptors IL-2Rα, IL-2Rβ, and IL-2Rγ. IL-2Rβ recruits JAK-1, while JAK-2 is recruited by IL-2Rγ, and further phosphorylation of these proteins recruits several subtypes of STAT molecules, mainly STAT-1, STAT-3, STAT-5a, and STAT-B. Furthermore, activation of Pi3K and MAP-k results in the activation of the SHC proteins, which end up with mTOR signaling, promoting the process of inflammation and cell proliferation (65). At the wound site, IL-2 activates macrophages and NK cells and promotes the proliferation of B-lymphocytes and T-lymphocytes. IL-2 plays a crucial role in THC activation and is involved in the development and activation of TH-1 and TH-2, which are necessary for cutaneous wound healing (66).

The current treatment modalities involving IL-2 as a target are used to treat several carcinomas, such as melanoma and renal cell carcinoma (67). The route of administration of IL-2-based targeted therapies also has an impact on treatment. It is evident that systemic therapies of IL-2 have a narrow therapeutic index, and the ideal route of administration of IL-2-based drug therapies is usually intralesional as it is more effective (68). Decreased IL-2 signaling has been reported in patients with Diabetes mellitus due to poor phosphorylation of STAT-5 in the IL-2 signaling cascade as well as single nucleotide polymorphisms (SNP), which can eventually result in aberrant wound healing. IL-2 receptor-mediated targeted therapy will be helpful as it has been observed to be a potential target for wound healing in Diabetes (68).

## Role of non- interleukin cytokines

TNF-α, TGF-β, and CRP are non-interleukin cytokines that also play a role in wound healing. TNF-α is a pro-inflammatory cytokine that, when present in lower concentrations, can initiate the process of inflammation in the inflammatory phase of wound healing (69). On the other hand, if present in higher concentrations, it can inhibit the process of wound healing by degrading the extracellular matrix formation in the remodeling phase of wound healing. TNF-α is synthesized mainly by macrophages, other than lymphocytes and adipose tissues. TNF-α, in turn, binds with TNFR-2 and activates the MAP-K and NF-Kβ signaling cascade, promoting the process of wound healing (70).

TGF-β is responsible for the recruitment of neutrophils and monocytes early in the process of wound healing (71). Besides this, it also plays a crucial role in the differentiation of monocytes into macrophages. Consequently, the formation of fibroblasts begins with the process of proliferation, resulting in the formation of an extracellular matrix that promotes wound healing (72). Patients with Diabetes mellitus show a reduction in TGF-β expression, and impaired TGF signaling delays the process of wound healing (32). CRP is one of the most common non-interleukin cytokines that serves as a pattern recognition receptor and increases the production of pro-inflammatory cytokines such as IL6, TNF-α, and IL-1ß, which are necessary for wound healing, mainly in the inflammatory phase of wound healing (73). Patients with Diabetes mellitus have elevated CRP levels in their blood, which can be used as a diagnostic marker in non-healing diabetic wounds (**Table 1**).

**Table 1:** Role of pro-inflammatory and anti-inflammatory cytokines in wound healing.

S.No	Phases in Wound Healing	Pro-inflammatory Cytokines	Physiological role	Anti-inflammatory Cytokines	Physiological Role
1.	**Haemostasis** **Phase**	IL-1ß TNF-α IL-6 IL-8	• Initiate inflammatory response. • Promote vasodilation and increased vascular Permeability.	IL- 10 TGF-ß	• Suppress pro-inflammatory response. • Modulate extracellular matrix production.
2.	**Inflammatory Phase**	IL-1ß TNF-α IL-6 IL-8 IL-12	• Recruit immune cells to the wound site. • Enhance phagocytosis and clearance of debris. • Activate fibroblasts for tissue repair.	IL- 10 TGF-ß	• Limit excessive inflammation. • Promote angiogenesis. • Stimulate collagen synthesis.
3.	**Proliferative Phase**	IL-1ß TNF-α IL-6 IL-8	• Promote angiogenesis. • Induce proliferation and migration of Fibroblasts and Keratinocytes.	IL- 10 TGF-ß	• Stimulate fibroblast proliferation and matrix remodeling. • Support tissue remodeling.
4.	**Remodeling Phase**	IL- 10 TGF-ß	• Control inflammatory response • Modulate extracellular matrix remodeling	IL-1ß TNF-α IL-6 IL-8	• Resolve persistent inflammation. • Promote tissue maturation and stability.

## Conventional approaches in wound healing

Diabetes mellitus complications like diabetic foot ulcers are exceedingly costly and challenging to cure. There are numerous topical agents indicated to treat this condition. Magistral preparations are frequently blended to tailor a therapy for a particular patient’s problem. This enables the patient to receive treatment that has been customized just for them. This Magistral Preparation of topical antimicrobial agents such as silver nitrate blended with a Peru balsam obtained from Myroxylon pereirae resin has a promising wound healing activity against non-healing diabetic wounds. Since ancient times, silver has been utilized for treating ailments. Silver-impregnated dressings appear to prevent or minimize the risk of amputations in a patient, as silver possesses excellent Antimicrobial Properties. Peru balsam proved to be an excellent antiseptic and antibacterial effect. For the rapid wound healing activity, Glycosaminoglycan derivatives, notably sulodexide, can be added to the Magistral preparations (74).

Despite the availability of several systemic antibiotics, topical antimicrobial treatment is widely used as it has several advantages; it may prevent the use of systemic antibiotics and thereby prevent antimicrobial resistance and improves patient compliance/adherence to the therapy and, most importantly, compared to systemic therapy, the risk of medication toxicity is significantly lower. In the normal course of wound healing, collagen develops, and the precise function of antiseptics is unveiled. Conventionally used Antiseptics are sodium hypochlorite and hexachlorophene, which are not widely used nowadays. The optimal topical agents should be formulated such that they possess antimicrobial capabilities, notably antibacterial activity against numerous gram-positive and gram-negative bacteria, as the wound region is susceptible to infectious pathogens (75). The commonly used topical formulations for treating chronic wounds are described in **Table 2**.

**Table 2:** Miscellaneous interleukins in wound healing.

S.No	Interleukins	Source	Physiological Function
1.	**Interleukin – 23**	Macrophages and dendritic cells	• IL-23 prompts T cells to continue generating IL-17 by acting on them. Similar signaling pathways can be activated by IL-23 and IL-12; however, IL-23 only slightly activates the STAT4 proteins involved in inflammation. • Additionally, pro-inflammatory cytokines, including IL-1, -6, and TNF- are stimulated by the autocrine/paracrine actions of IL-23 on macrophages underlining IL-23’s role in the inflammatory process (76).
2.	**Interleukin - 4**	CD4+T cells (Th2)	• IL-4 influences both B and T cells. It is a B-cell proliferation factor that influences the selection of IgE and IgG1 isotypes. • It promotes Th2 differentiation and proliferation and prevents macrophage activation induced by IFN gamma. By lowering the production of fibronectin, IL-4 hindered the keratinocyte’s response to wound healing (77).
3.	**Interleukins – 13**	CD4+T cells (Th2), N.K.T. cells and mast cells	• It influences B cells, epithelial cells, fibroblasts, monocytes, and fibroblasts. Significant effects of IL-13 include stimulation of IgE isotype flipping and B-cell proliferation and differentiation. • Increased collagen synthesis by fibroblasts, increased mucus production by epithelial cells, and inhibition of pro-inflammatory cytokine production are all results of IL-13 activation. Additionally, IL-13 and IL-4 work alongside the biological effects on inflammation & wound healing (78).
4.	**Interleukin - 31**	Th2 cells and dendritic cells.	• IL-31 stimulates the production of chemokines and the synthesis of IL-6, IL-16, and IL-32. • IL-31 aids in generating cell-mediated defenses against infections. It has also been noted to play a significant role in some chronic inflammatory conditions where there is delayed wound healing (69).
5.	**Interleukin - 33**	Mast cells and Th2 lymphocytes	• IL-33, influences a wide range of innate and immunological cells, including dendritic cells, T lymphocytes, and B lymphocytes. The cells seen in barrier areas have high levels of interleukin (IL)-33, which uses the ST2 receptor to transmit signals. • After acute inflammation, IL-33 signaling *via* ST2 is crucial for tissue homeostasis, supporting fibrinogenesis and wound repair at damage sites (79).

There are more non-healing wound forms that mirror non-healing diabetic wounds in several ways, mainly Peri -ulcers and vascular ulcers. The tissue surrounding an injury is known as the peri ulcers (also peri-wound). The typical peri-wound area limit is 4 cm, although it may go beyond that if there is skin injury on the exterior of the wound. Before a wound treatment plan is recommended, the peri-wound evaluation is a crucial stage in the wound assessment process. Burning, itching, discomfort, and pain are common in peri-wound patients. Moisture barriers (ointments, salves, and films), topical corticosteroids, antiseptics, and antifungal agents, as well as moisture-regulating dressings like self-adaptive wound dressing, may be used as part of local treatment to protect the peri-wound and maintain its healthy functionality (80).

When the leg veins fail to properly push blood back up to your heart, venous ulcers (open sores) can develop. Pressure rises in the veins as a consequence of blood clotting. The untreated increased pressure and surplus fluid in the damaged region might result in an open sore; this entire process is collectively known as chronic venous insufficiency. The majority of venous ulcers develop on the leg, just above the ankle. This type of wound could take an extended period to heal completely (81).

## Novel approaches in chronic wound healing

Wounds are considered chronic if there is a disruption of the normal wound-healing process, resulting in impaired wound healing for at least three months. Diabetic foot ulcers and non-healing pressure ulcers are among the most common types of chronic wounds. Diabetic wounds are particularly detrimental due to changes in the capillary system, such as thickening of the basement membrane, reduction in the size of the capillary vessel, and alterations in glucose levels, which may lead to vasoconstriction and ischemia, making the wound worse. Non-healing ulcers are a significant global burden, affecting over 40 million people per year. The most typical symptoms of chronic wounds are a prolonged inflammatory phase, phenotypic anomalies, including the expression of wound-healing proteins, and impaired formation of the extracellular matrix (82).

The application of different biomaterials and additive manufacturing technology in wound healing has gained significant attention in recent years. These approaches offer innovative solutions to enhance wound healing processes, improve treatment outcomes, and address the challenges associated with traditional wound management. Biomaterials for Wound Dressings, including hydrogels, films, foams, and nanofibers, are used to develop advanced wound dressings. These dressings can provide a moist wound environment, facilitate gas exchange, absorb exudate, and protect the wound from external contaminants. Incorporation of bioactive molecules, such as growth factors, antimicrobial agents, and extracellular matrix components, into the dressings can promote wound healing processes. Scaffold-based Tissue Engineering methods like Biomaterial scaffolds offer a three-dimensional (3D) framework to support cell adhesion, migration, and tissue regeneration. Biomaterial-based skin substitutes and dermal matrices have been developed to promote wound healing and tissue regeneration. These constructs provide a temporary or permanent replacement for damaged or lost skin tissue. They can support cell attachment, proliferation, and differentiation and may include bioactive factors to stimulate angiogenesis and collagen synthesis. Biomaterials can be engineered with bioactive coatings or nanoparticles to provide localized drug delivery systems. These coatings or nanoparticles can release therapeutic agents, growth factors, or antimicrobial agents in a controlled and sustained manner, promoting wound healing and preventing infections. Biomaterials and additive manufacturing techniques can be utilized to develop wearable devices or sensors for real-time monitoring of wound healing parameters. These devices can measure parameters like temperature, pH, moisture, oxygen levels, and biomarker concentrations, providing valuable insights into wound healing progression and enabling timely interventions. The application of biomaterials and additive manufacturing technology in wound healing holds great potential to revolutionize wound care by providing tailored and advanced solutions. These approaches aim to accelerate healing, reduce complications, enhance patient comfort, and improve overall outcomes in the management of acute and chronic wounds. Continued research and development in this field are vital to further advance these technologies and bring them into clinical practice.

Macrophage polarization profiling refers to the characterization and classification of macrophages based on their functional properties and behavior. Macrophages are immune cells that play a crucial role in tissue healing and regeneration. They can exhibit different phenotypes or polarization states, broadly classified as pro-inflammatory (M1) or anti-inflammatory (M2). Native and regenerated silk biomaterials are biocompatible materials that have been used in various biomedical applications, including tissue engineering and drug delivery. Understanding how macrophages interact with these biomaterials and how they influence macrophage polarization is important for optimizing their performance and therapeutic outcomes. In a study focusing on macrophage polarization profiling on native and regenerated silk biomaterials, researchers aimed to investigate how these biomaterials modulate macrophage behavior and polarization. The study involved evaluating the response of macrophages to silk biomaterials and characterizing their polarization states. To perform the profiling, macrophages were cultured on the native and regenerated silk biomaterials. Various assays and techniques were employed to assess macrophage polarization, such as gene expression analysis, cytokine secretion profiling, and surface marker analysis. These methods provided insights into the specific polarization states and functional characteristics of macrophages on silk biomaterials. The results of the study shed light on how native and regenerated silk biomaterials influence macrophage polarization. Understanding the macrophage polarization profile on native and regenerated silk biomaterials has implications on the design and development of silk-based biomaterials for tissue engineering and other biomedical applications (83).

The regulation of decellularized matrix-mediated immune response refers to the control and modulation of the immune system’s reaction when exposed to decellularized matrices. Decellularized matrices are natural or synthetic materials that have had their cellular components removed, leaving behind the extracellular matrix (ECM) structure. In the context of tissue engineering and regenerative medicine, decellularized matrices are used as scaffolds to support tissue regeneration and repair. However, when these matrices are implanted in the body, they can trigger an immune response. The immune response to decellularized matrices can vary, and it is desirable to regulate this response to promote tissue integration and prevent adverse reactions. Several strategies are employed to achieve immune regulation in decellularized matrix-mediated responses. One approach involves modifying the decellularized matrices to reduce the presence of immunogenic components and increase their biocompatibility. This can be achieved through various methods, such as optimizing the decellularization process, treating the matrices with enzymes or chemicals, or incorporating immunomodulatory factors into the matrices. Another strategy focuses on modulating the immune cells behavior and response to the decellularized matrices. This can be done by incorporating specific molecules or factors into the matrices that promote an anti-inflammatory or immune-tolerant environment. These factors can include growth factors, cytokines, or immunomodulatory agents. Overall, the regulation of decellularized matrix-mediated immune response involves modifying the matrices themselves to reduce immunogenicity, incorporating immunomodulatory factors, and controlling the release of bioactive molecules. These approaches aim to promote tissue integration, minimize inflammation, and improve the overall success of tissue engineering and regenerative medicine therapies using decellularized matrices (84).

Functional hydrogels have emerged as promising materials for diabetic wound management. Diabetic wounds are a significant complication of diabetes and often exhibit impaired healing due to factors such as reduced blood flow, chronic inflammation, and high glucose levels. Functional hydrogels offer unique properties that can address these challenges and promote wound healing. In recent advancements, functional hydrogels have been developed with specific characteristics tailored for diabetic wound management. These hydrogels possess attributes such as high-water content, biocompatibility, flexibility, and controlled release capabilities (**Table 3**). One key feature of functional hydrogels is their ability to maintain a moist environment at the wound site, which is crucial for optimal healing (85). They can absorb excess wound exudate while providing moisture to the wound bed, promoting cell migration, and facilitating the formation of new tissue. Functional hydrogels also exhibit bioactive properties by incorporating growth factors, antimicrobial agents, and extracellular matrix components. These bioactive components can enhance cell proliferation, angiogenesis, and collagen synthesis, thereby accelerating wound closure and tissue regeneration. Functional hydrogels can modulate the inflammatory response in diabetic wounds. By incorporating anti-inflammatory agents or immune-regulatory molecules, these hydrogels can help mitigate chronic inflammation commonly observed in diabetic wounds and create an environment conducive to healing. Additionally, functional hydrogels can be engineered to provide controlled drug release. They can deliver therapeutic agents such as growth factors, antibiotics, or wound healing-promoting compounds directly to the wound site, ensuring sustained and localized treatment. These hydrogels hold great potential in improving the healing outcomes of diabetic wounds by addressing the specific challenges associated with diabetes-induced impaired wound healing (86).

**Table 3:** Different topical agents used in the treatment of non-healing diabetic ulcers.

S.No	Topical Agents	Formulation	Antimicrobial Spectrum	Advantages/Benefits
1.	**Acetic Acid**	Solution (0.5%, 0.25%, 0.1%)	Bactericidal against more gram-positive and gram-negative bacteria – Pseudomonas aeruginosa	Inexpensive and most widely used in wound healing.
2.	**Cetrimide**	Solution (40%)	Active against many bacteria and fungi	Exhibit excellent antiseptic action along with antimicrobial properties.
3.	**Chlorhexidine gluconate**	• Solution (2%, 4%) • Sponges • Wipes	Active against gram-positive bacteria (Staphylococcus aureus) and gram-negative (Pseudomonas aureus)	It exhibits a half-life of 6 hours and acts as a potential antiseptic agent.
4.	**Hexachlorophene**	• Liquid (3%) • Foam (0.23%)	Bacteriostatic against staphylococcus species and other gram-positive bacteria.	It has more retention capacity in the wound site and potentiates the wound-healing process.
5.	**Povidone Iodine**	• Ointment (1%) • Solution (10%)	Broad Spectrum against Staphylococcus aureus and Enterococci.	Potent wound healing activity and anti-allergic.
6.	**Silver Nitrate**	• Solution (0.5%, 10%, 25%) • Ointment (10%)	Silver ions are bactericidal against a broad spectrum of gram-positive and gram-negative bacteria.	Economical and widely used
7.	**Hydrogen Peroxide**	• Solution (1%, 3%) • Cream (1%)	The oxidizing agent, active against many gram-positive and gram-negative bacteria	Easily applicable and economical, widely used in cleansing the wound area.

The current treatment of wounds focuses more on treating their etiology rather than conventional wound care, which involves wound debridement and the use of pre-existing dressings like gauze-woven cotton composite dressings due to their ease of use and cost-effectiveness. However, these conventional dressings have limitations, such as adherence to the wound bed and the presence of moisture, which can be addressed with newer synthetic dressings that provide comfort and promote wound healing. The most advanced wound healing formulations available include film, foam, hydrogel, and hydrocolloids, which can be incorporated into conventional dressing materials. Hydrogels have promising wound healing potential in terms of exudate absorption, gas exchange from the wound site, and adherence to the wound bed (87). Newer therapeutic approaches aim to incorporate additives into present dressing materials, including antimicrobial molecules and immunomodulatory cytokines. While biological dressings can interact with the matrix in the wound bed to speed up the healing process, their use is limited due to decreased interaction with the wound tissue. Nanoparticles have a promising role in wound healing, both in terms of drug delivery and intrinsic activity (88).

Nanofibers are fine filaments produced by the process of electrospinning, which is economical and simple. Nanofiber meshes have a significant role in the treatment of wounds, as they partially replicate the extracellular matrix and support wound healing by maintaining moisture in the wound site, preventing wound dehydration and microbial contamination. Nanofibers can be natural polymers, polysaccharides, or proteins (89). The adoption of these technologies and the establishment of novel therapeutic interventions are difficult due to a gap in our complete understanding of the pathophysiological mechanisms at the cellular and molecular level, as well as a lack of data assessing safety and bioavailability differences among individual patients.

## Conclusion

Diabetes and its complications remain a global burden, causing a significant increase in mortality and morbidity each year, as reported by WHO’s health statistics. Even with the utmost care and disease-specific rational drug therapy, patients may still end up with permanent disabilities such as blindness, amputation, and renal failure. Treating diabetic wounds remains challenging due to the distortion in the tissue repair mechanism and the continuous cutaneous wounds that attract infectious pathogens to invade the wound tissue and form a colony. While surgical intervention by wound debridement is the gold standard and a better therapeutic option for treating diabetic wounds, non-invasive approaches are needed.

The concept of studying the underlying molecular and cellular level mechanisms of wound healing to formulate better therapeutic outcomes has emerged. The complicated process of wound healing is hampered by Diabetes, as shown in **Figure 2**, which also illustrates how the normal wound healing processes are affected. The initial stage of wound healing involves recruiting many inflammatory cells, such as cytokines, neutrophils, and chemokines. However, this process of recruitment is delayed in diseases like Diabetes. Pro-inflammatory cytokines play a crucial role in initiating wound healing, where there is an upregulation of IL-1ß, TNF-α, IL-6, TGF-ß, and CRP and a downregulation of IL-10. This leads to an unfavorable wound environment for the healing process. Rather than superficially treating diabetic wounds conventionally, which paves the way for re-infections, target-specific pro-inflammatory cytokines-based therapies, either by upregulation or downregulation of them, can be helpful in the wound healing process. This can enhance the quality of life in patients, which is the goal of drug therapy.

## Author contributions 

Conceptualization, SN, JN, TT, and VS; Resources, VC, GG, NF, MS, SF, GR, SS, LW, and SN; writing—original draft preparation, SN, JN, TT, VS, and VC; writing—review and editing, SN, JN, TT, and VS. All authors contributed to the article and approved the submitted version.
